# Analysis of multivariate longitudinal kidney function outcomes using generalized linear mixed models

**DOI:** 10.1186/s12967-015-0557-2

**Published:** 2015-06-14

**Authors:** Miran A Jaffa, Mulugeta Gebregziabher, Ayad A Jaffa

**Affiliations:** Epidemiology and Population Health Department, Faculty of Health Sciences, American University of Beirut, P.O. Box 11-0236, Riad El-Solh, Beirut, 1107 2020 Lebanon; Department of Public Health Sciences, Medical University of South Carolina, Charleston, SC 29425 USA; Department of Medicine, Medical University of South Carolina, Charleston, SC USA; Department of Biochemistry and Molecular Genetics, Faculty of Medicine, American University of Beirut, P.O. Box 11-0236, Riad El-Solh, Beirut, 1107 2020 Lebanon

**Keywords:** Joint modeling, Multivariate longitudinal outcomes, Random coefficients

## Abstract

**Background:**

Renal transplant patients are mandated to have continuous assessment of their kidney function over time to monitor disease progression determined by changes in blood urea nitrogen (BUN), serum creatinine (Cr), and estimated glomerular filtration rate (eGFR). Multivariate analysis of these outcomes that aims at identifying the differential factors that affect disease progression is of great clinical significance. Thus our study aims at demonstrating the application of different joint modeling approaches with random coefficients on a cohort of renal transplant patients and presenting a comparison of their performance through a pseudo-simulation study. The objective of this comparison is to identify the model with best performance and to determine whether accuracy compensates for complexity in the different multivariate joint models.

**Methods and results:**

We propose a novel application of multivariate Generalized Linear Mixed Models (mGLMM) to analyze multiple longitudinal kidney function outcomes collected over 3 years on a cohort of 110 renal transplantation patients. The correlated outcomes BUN, Cr, and eGFR and the effect of various covariates such patient’s gender, age and race on these markers was determined holistically using different mGLMMs. The performance of the various mGLMMs that encompass shared random intercept (SHRI), shared random intercept and slope (SHRIS), separate random intercept (SPRI) and separate random intercept and slope (SPRIS) was assessed to identify the one that has the best fit and most accurate estimates. A bootstrap pseudo-simulation study was conducted to gauge the tradeoff between the complexity and accuracy of the models. Accuracy was determined using two measures; the mean of the differences between the estimates of the bootstrapped datasets and the true beta obtained from the application of each model on the renal dataset, and the mean of the square of these differences. The results showed that SPRI provided most accurate estimates and did not exhibit any computational or convergence problem.

**Conclusion:**

Higher accuracy was demonstrated when the level of complexity increased from shared random coefficient models to the separate random coefficient alternatives with SPRI showing to have the best fit and most accurate estimates.

## Background

Monitoring kidney function is a necessity for patients who underwent kidney transplantation to assess progression of renal disease. Typically three markers are measured repeatedly over time after transplantation to ensure that no signs of kidney problems or risk of renal failure due to graft rejection are present and these include blood urea nitrogen (BUN), serum creatinine (Cr) and estimated glomerular filtration rate (eGFR). These markers are correlated and needed to ensure an accurate evaluation of the kidney function since each has its own limitations and could be influenced by demographical and physiological characteristics of the patient [1–5]. Once a patient experiences graft rejection then measurements on the kidney markers won’t be recorded and patients will no longer be monitored for kidney function resulting in missing data. Given the interdependence of these outcomes in determining kidney function, it is important to evaluate the factors that affect the rate of change in these outcomes in a joint manner. The motivation of this study is to assess the association between the three outcomes (BUN, creatinine and eGFR) and key demographic and clinical factors accounting for the correlation between these markers in a multivariate fashion.

Analysis of multiple outcomes is given special attention in the literature. For example, multivariate linear mixed models (MLMM) were proposed to analyze multiple outcomes to assess and test for global exposure effect across outcomes while assuming flexible correlation structure for the multiple outcomes [6]. The idea was to provide robust estimates for the mean by separating it from the correlation parameters. This MLMM model was limited to Gaussian outcomes and did incorporate longitudinal data. Joint modeling was also used in the context of jointly studying time to clinical event and repeated measures on surrogate outcomes [7]. Others included joint modeling of the multilevel item response theory (MLIRT) and Cox’s proportional hazard model for time to dependent terminal event with shared random effects to link the two models [8]. A modeling framework for MLIRT also referred to as latent regression was widely considered and was based on the idea that the observed measurements are a result of some imperfect interaction between subject-specific latent variables and measurement-specific parameters [9–13]. The latent traits are considered as response variables and are regressed on a set of covariates, hence the name of latent regression. The advantage of the MLIRT models resides in the separation of the measurement-specific parameters and subject-specific covariates from manifest data and simultaneous estimation of these parameters and covariate effects [14–16]. However, deviation from the normality assumption could affect the accuracy of the estimates and the inferences for the high-level outcomes [17]. Multivariate joint modeling was also an alternative wherein a joint distribution is specified to jointly model all random effect [18–20]. Despite that data transformation solved the issue of non-normality it still had its imbedded disadvantages such as difficulty in the interpretation of the estimated parameters on transformed scale. In addition, joint normality may not be always achieved especially if transformation was conducted on a component basis, and lack of universal transformation that could be appropriate for the majority of datasets [21]. An alternative approach that is not constrained by the issue of non-normality is the joint modeling of multivariate longitudinal outcomes discussed in [22]. In this approach the interdependence among outcomes is captured by using shared latent variable(s) or the correlation between random coefficients corresponding to each outcome and the non-normality is solved using distributions and link functions from the list of general linear models. They describe the following four approaches, (1) shared random intercept (SHRI); (2) shared random intercept and slope (SHRIS); (3) separate random intercepts (SPRI); (4) separate random intercepts and slopes (SPRIS). The application and assessment of performance for these models were limited to health services outcomes in extremely large datasets and were not applied on clinical outcomes from a relatively small sample size.

The goals of our study were

1. To identify the best fitting joint model from the four approaches listed in terms of the tradeoff between the complexity of the model and precision of the estimates including convergence related complexities that could be due to small sample size.
2. To demonstrate the application of these models on a cohort of renal transplant patients assuming that the three correlated kidney markers follow the lognormal distribution. Hence in this research work we are proposing a novel application of the joint modeling approaches, and we are conducting an innovative thorough assessment of the performance of each approach in a clinical setting characterized by its small sample size. Studies with small sample sizes are commonly encountered in clinical settings of high cost, or where focus is on rare diseases, pilot studies as well as basic science research. Hence demonstrating the application and understanding the performance of these models in a dataset characterized by its small sample size, and multiple outcomes with non-normal distribution and high correlation is the key novel feature of this study.

## Methods

The joint modeling approach investigated in this study is the multivariate generalized linear mixed models (mGLMM) which is a generalization of the linear mixed models. Fixed and random effects are both included and are referred to as $\beta$ and b_i_ respectively in the below equations of the joint models. Fixed effects represent the average rate of change in the outcome attributed to specific covariates at a population level; however, the random effects represent subject specific rate of change. The correlation between repeated measures on a certain outcome pertaining to the same individual subject is accounted for through the random effects. The correlations between the different multiple outcomes are incorporated through the variance–covariance matrix of the shared or separate random effects depending on the specific mGLMM. While linear mixed models are limited to normally distributed outcomes, mGLMM are extended to multiple correlated outcomes with other distributions, not necessarily normal, each of which is determined by its corresponding link function. The mGLMM is comprised of a set of generalized linear mixed models (GLMM) linked together by a SHRI, SHRIS, SPRI or separate random intercept and slope (SPRIS).

Let Y_ij1_, Y_ij2_,…, Y_ijk_ be the multivariate outcomes for subject i, measured at times j = 1 to t_i_, for outcomes 1 to k, the different joint models can be described as such:

## SHRI

For three outcomes, SHRI model can be described as follows:

$$ \begin{aligned} E\left( {Y_{ij1} |b_{0i} } \right) = g^{ - 1} \left( {\beta_{01} + b_{0i} + \beta_{11} t_{i} + \beta_{21} x_{i} } \right) \hfill \\ E\left( {Y_{ij2} |b_{0i} } \right) = g^{ - 1} \left( {\beta_{02} + b_{0i} + \beta_{12} t_{i} + \beta_{22} x_{i} } \right) \hfill \\ E\left( {Y_{ij3} |b_{0i} } \right) = g^{ - 1} \left( {\beta_{03} + b_{0i} + \beta_{13} t_{i} + \beta_{23} x_{i} } \right) \hfill \\ \end{aligned} $$

where $b_{0i} \sim MVN\left( {0,\sigma_{b}^{2} } \right)$, $\sigma_{b}^{2}$ is the variance for the intercept, $x_{i}$ is the covariate of interest and $g^{ - 1}$ is the link function. The model can accommodate more than one covariate. The correlation between the outcomes is assumed to be captured by the shared-common intercept.

## SHRIS

$$ \begin{aligned} E\left( {Y_{ij1} |b_{i} } \right) = g^{ - 1} \left( {\beta_{01} + b_{0i} + \left( {b_{1i} + \beta_{11} } \right)t_{i} + \beta_{21} x_{i} } \right) \hfill \\ E\left( {Y_{ij2} |b_{i} } \right) = g^{ - 1} \left( {\beta_{02} + b_{0i} + \left( {b_{1i} + \beta_{12} } \right)t_{i} + \beta_{22} x_{i} } \right) \hfill \\ E\left( {Y_{ij3} |b_{i} } \right) = g^{ - 1} \left( {\beta_{03} + b_{0i} + \left( {b_{1i} + \beta_{13} } \right)t_{i} + \beta_{23} x_{i} } \right) \hfill \\ \end{aligned} $$

$$ b_{i} = \left( {\begin{array}{*{20}c} {b_{0i} } \\ {b_{1i} } \\ \end{array} } \right) \sim MVN\left( {0,D_{2 \times 2} } \right) $$ where $D$ is the variance–covariance matrix of the shared intercept and slope so intuitively it includes the variances of the intercept and slope and their covariance. The correlation between the outcomes is assumed to be captured by the shared-common intercept and slope.

## SPRI

The concept of capturing the correlation between the outcomes through intercept only, still applies here as in SHRI but the difference is in relaxing the shared intercept theory and assuming separate intercept associated with every outcome. Relaxing this assumption adds a layer of complexity by increasing the number of parameters to be estimated in the variance–covariance matrix. SPRI can be described as such

$$ \begin{aligned} E\left( {Y_{ij1} |b_{0i} } \right) = g^{ - 1} \left( {\beta_{01} + b_{0i1} + \beta_{11} t_{i} + \beta_{21} x_{i} } \right) \hfill \\ E\left( {Y_{ij2} |b_{0i} } \right) = g^{ - 1} \left( {\beta_{02} + b_{0i2} + \beta_{12} t_{i} + \beta_{22} x_{i} } \right) \hfill \\ E\left( {Y_{ij3} |b_{0i} } \right) = g^{ - 1} \left( {\beta_{03} + b_{0i3} + \beta_{13} t_{i} + \beta_{23} x_{i} } \right) \hfill \\ \end{aligned} $$

$$ b_{0i} = \left( {\begin{array}{*{20}c} {b_{0i1} } \\ {b_{0i2} } \\ {b_{0i3} } \\ \end{array} } \right) \sim MVN\left( {0,D_{3 \times 3} } \right) $$ where the number of parameters to be estimated increases to 6 attributed to the 3 variances and 3 covariances between the intercepts. The correlation between the 3 outcomes is assumed to be captured by the correlation between the separate intercepts.

## SPRIS

The concept of capturing the correlation between the outcomes as in SHRIS is applied here with the difference in assuming separate intercept and slope for every outcome which adds another level of complexity on SPRI. SPRIS can be described as such:

$$ \begin{aligned} E\left( {Y_{ij1} |b_{i1} } \right) = g^{ - 1} \left( {\beta_{01} + b_{0i1} + \left( {b_{1i1} + \beta_{11} } \right)t_{i} + \beta_{21} x_{i} } \right) \hfill \\ E\left( {Y_{ij2} |b_{i2} } \right) = g^{ - 1} \left( {\beta_{02} + b_{0i2} + \left( {b_{1i2} + \beta_{12} } \right)t_{i} + \beta_{22} x_{i} } \right) \hfill \\ E\left( {Y_{ij3} |b_{i3} } \right) = g^{ - 1} \left( {\beta_{03} + b_{0i3} + \left( {b_{1i3} + \beta_{13} } \right)t_{i} + \beta_{23} x_{i} } \right) \hfill \\ \end{aligned} $$

$$ b_{i} = \left( {\begin{array}{*{20}c} {b_{i1} } \\ {b_{i2} } \\ {b_{i3} } \\ \end{array} } \right) = \left( {\begin{array}{*{20}c} {\left( {\begin{array}{*{20}c} {b_{0i1} } \\ {b_{1i1} } \\ \end{array} } \right)} \\ {\left( {\begin{array}{*{20}c} {b_{0i2} } \\ {b_{1i2} } \\ \end{array} } \right)} \\ {\left( {\begin{array}{*{20}c} {b_{0i3} } \\ {b_{1i3} } \\ \end{array} } \right)} \\ \end{array} } \right) \sim MVN\left( {0,D_{6 \times 6} } \right) $$ where D is the variance–covariance matrix for the 3 intercepts and 3 slopes associated with every outcome resulting in an increase of the number of parameters to be estimated to 21. For all the models, the dependence between and within outcomes is captured by the structure of the variance–covariance matrix that can be determined by the measures of model fitting AIC, BIC and −2log l.

The univariate counterpart of these models can be reduced to GLMM with random intercept only (RI) and with random intercept and slope (RIS).

## Pseudo-simulation bootstrap study

To assess the performance of each model a pseudo simulation bootstrap study was conducted using a renal dataset and assuming that the three kidney outcomes (BUN, Cr and eGFR) follow the lognormal distribution. The covariates were limited to time and square of time since SPRIS exhibited convergence problems when other covariates were included. The square of time was included in the analysis to account for the curvilinear relationship between time and each of the outcomes. We implemented 2,000 replications each with a sample size of 110 individuals boot strapped from the cohort of renal patients. The performance of the 4 multivariate models (SHRI, SHRIS, SPRI, SPRIS) and that of the univariate random intercept model (RI), and random intercept and slope model (RIS) was examined. The performance was determined in terms of accuracy assessed by two measures; the mean of the differences between the estimates of the bootstrapped datasets and the true beta obtained from the application of each model on the renal dataset, and the mean of the square of these differences. Specifically, true betas are the fixed effects $\left( {\beta_{11} ,\beta_{12} ,\beta_{13} ,\beta_{21} ,\beta_{22} ,\beta_{23} } \right)$ in the models’ equations. These parameters are estimated by applying the generalized linear mixed models on the renal transplant dataset to reflect the estimated effects of the corresponding covariates on the changes in kidney outcomes. In Table 1, the differences between the estimates generated in the simulation studies and these betas are computed to determine the accuracy of the various models, noting that the smaller the difference the more accurate the model. For simplicity, we refer to these measures as mean difference, and mean squared difference respectively. This comparison helped us achieve the following objectives:

**Table 1:** Bootstrapping on the (a) multivariate models and (b) univariate models: replicate 2,000 sample size 110

Estimate	Outcome	(a) Multivariate models									(b) Univariate models								
		SHRI			SHRIS			SPRI^d^			SPRIS			RI			RIS		
		True beta^a^	Mean difference^b^	Mean squared difference^b^	True beta	Mean difference	Mean squared difference	True beta	Mean difference	Mean squared difference	True beta	Mean difference	Mean squared difference	True beta	Mean difference	Mean squared difference	True beta	Mean difference	Mean squared difference
Intercept	BUN*100^c^	3.3899	−0.09021	0.07796	3.3887	−0.10200	0.07740	3.3938	−0.08663	0.08125	3.3895	−0.12931	0.08517	3.3914	−0.08466	0.07908	3.3887	−0.10027	0.08003
	Creatinine*100	0.9405	−0.04489	0.12977	0.9394	−0.06113	0.12942	0.9461	−0.03864	0.13702	0.9422	−0.00693	0.13821	0.9416	−0.04614	0.13064	0.9411	−0.04301	0.13186
	eGFR*100	3.3728	0.13339	0.13729	3.3716	0.14946	0.13749	3.3626	0.11698	0.15495	3.3655	0.05496	0.16161	3.3674	0.11982	0.14741	3.3687	0.06382	0.15037
Time	BUN*10^3^	−0.0195	0.01402	0.00458	−0.0193	0.01465	0.00458	−0.0186	0.03321	0.00451	−0.01857	0.11809	0.00509	−0.0191	0.02583	0.00456	−0.0188	0.01559	0.00462
	Creatinine*10^4^	−0.0452	−0.46142	0.04717	−0.0451	−0.42203	0.04633	−0.0441	−0.07492	0.04227	−0.04421	−0.52669	0.04300	−0.0452	−0.32036	0.04663	−0.0452	−0.42299	0.04670
	eGFR*10^3^	0.0532	0.10479	0.00853	0.0534	0.10473	0.00880	0.0503	0.01589	0.00576	0.05049	0.03443	0.00610	0.0518	0.05929	0.00648	0.0518	0.04602	0.00650

^a^True beta obtained from the application of every model on the renal transplant dataset.

^b^Mean difference and mean squared difference represent respectively the average deviation and its square of every bootstrap estimate from the true value beta.

^c^Mean difference and mean squared difference should be multiplied by the number specified next to the outcome name.

^d^SPRI model has best results compared to other models: 50% of the estimates have smallest mean difference and mean squared difference under SPRI.

1. Identify the multivariate models that exhibited better performance than the other models.
2. Determine whether the complex separate random models (SPRI and SPRIS) had any advantage in terms of accuracy of the estimates over the simpler shared random effect models (SHRI and SHRIS). Accordingly, we can gauge complexity versus precision.
3. Examine whether including the slope as random effect along with the intercept as in the SHRIS and SPRIS models has any respective advantage over the models that assume solely random intercept as in SHRI and SPRI.
4. Evaluate whether there was a gain in accuracy under the multivariate modeling versus the univariate separate analysis implemented on every outcome.

The bootstrap results are presented in Tables 1, 2, and 3 for the multivariate and univariate implementation of the models with 2,000 replications. In Table 1 we present the mean difference and mean squared difference for the estimates under every model while in Table 2 we provide percent reductions in these measures for a specific multivariate model versus the others. In Table 3 the percent reductions in these measures between univariate and multivariate models are presented.

**Table 2:** Percent reduction in mean difference and mean squared difference that correspond to: SPRI compared to all other multivariate models (a, b, and c)^b^; SPRI compared to SHRI (d); SPRIS compared to SHRIS (e); SPRI compared to SPRIS(f)

	Estimate	Outcome	(a) SPRI vs SHRI	(b) SPRI vs SHRIS	(c) SPRI vs SPRIS	(d) SPRI vs SHRl^d^	(e) SPRIS vs SHRIS^e^	(f) SPRI vs SPRIS^f^
Mean difference	Intercept	BUN	4%	15%	33%	4%	(**–**)^c^	33%
		Creatinine	(**–**)^a^	(**–**)	(**–**)	14%	89%	(**–**)^c^
		eGFR	(**–**)	(**–**)	(**–**)	12%	63%	(**–**)
	Time	BUN	(**–**)	(**–**)	(**–**)	(**–**)^c^	(**–**)	96%
		Creatinine	84%	82%	86%	84%	(**–**)	86%
		eGFR	85%	85%	54%	85%	67%	74%
Mean squared difference	Intercept	BUN	(**–**)	(**–**)	(**–**)	(**–**)	(**–**)	5%
		Creatinine	(**–**)	(**–**)	(**–**)	(**–**)	(**–**)	1%
		eGFR	(**–**)	(**–**)	(**–**)	(**–**)	(**–**)	28%
	Time	BUN	2%	2%	11%	2%	(**–**)	11%
		Creatinine	10%	9%	2%	10%	7%	2%
		eGFR	32%	35%	6%	32%	31%	6%

^a^SPRI did not generate the smallest mean difference and/or mean squared difference compared to all multivariate models.

^b^50% of the estimates under SPRI had the smallest mean difference and mean squared difference compared to all other models with 58.7% and 12% average overall reduction respectively.

^c^No reduction was observed under SPRI or SPRIS.

^d^83% of the estimates under SPRI had smaller mean difference compared to SHRI with average overall reduction of 40%, and 50% had smaller mean squared difference with average reduction of 15%.

^e^50% of the estimates under SPRIS had smaller mean difference compared to SHRIS with average overall reduction of 73%, and 33% had smaller mean squared difference with average reduction of 19%.

^f^66% of the estimates under SPRI had smaller mean difference compared to SPRIS with average overall reduction of 72%, and 100% had smaller mean squared difference with average reduction of 9%.

**Table 3:** Percent reduction in mean difference that correspond to: SPRI Compared to RI (a); SPRIS compared to RIS (b)

Estimate	Outcome	Mean difference	
		(a) SPRI vs RI^b^	(b) SPRIS vs RIS^c^
Intercept	BUN	(–)^a^	(–)^a^
	Creatinine	16%	84%
	eGFR	2%	14%
Time	BUN	(–)	(–)
	Creatinine	77%	(–)
	eGFR	73%	25%

^a^No reduction was observed in mean difference under SPRI compared to RI or SPRIS compared to RIS.

^b^66% of the estimates under SPRI had smaller mean difference compared to RI with average overall reduction of 42%.

^c^50% of the estimates under SPRIS had smaller mean difference compared to RIS with average overall reduction of 41%.

*Objective 1* As evidenced in Tables 1 and 2 (see also 2nd footnote labeled as b in Table 2, and Table 2 columns a, b, and c), we notice that 6 out of 12 estimates i.e. 50% of the estimates generated under SPRI had the smallest mean difference, and mean squared difference compared to those generated under the other models that include SHRI, SHRIS, and SPRIS. Specifically, 50% of the estimates generated under SPRI had an associated mean difference, and mean squared difference that are respectively 58.7% and 12% smaller on average than those generated under the other models. Hence, this leads to the conclusion that SPRI demonstrated the best performance compared to the other 3 models.

*Objective 2* Compared to SHRI, 83% of the estimates generated under SPRI had associated mean difference that is 40% smaller on average, and mean squared difference that are close in value (15% reduction in mean squared differences under SPRI in 50% of the estimates) (see Table 2 column d, and footnote Table 2). In this context, we denote that the eGFR outcome had a significant 32% reduction in mean squared difference under SPRI compared to SHRI. When we compared SPRIS to SHRIS (see Table 2 column e, and footnote Table 2), we realized that about 50% of the estimates generated under SPRIS exhibited an approximate average reduction of 73% in mean difference compared to SHRIS; meanwhile, an observed reduction of 19% in mean squared difference was captured in only 33% of the estimates under SPRIS. Hence, mean squared difference did not greatly differ between SHRIS and SPRIS. Accordingly, one can conclude that separate modeling of the random effect had it be solely intercept (SPRI), or both intercept and slope (SPRIS) had an advantage specifically in mean difference reduction over the simpler models that assume shared random effects (SHRI and SHRIS).

*Objective 3* To assess whether the increase in complexity when going from the simpler model (SPRI) to the more complex one (SPRIS) generates more accurate results, we observed that 66% of the estimates under SPRI had an associated mean difference that is 72% smaller on average than those generated under SPRIS, and 100% of the estimates under SPRI had smaller mean squared difference (9% average reduction in mean squared difference) than those generated under SPRIS (see Table 2 column f, and footnote Table 2). Hence, the simpler model (SPRI) appeared to generate more accurate estimates than the complex model (SPRIS). This conclusion supports the finding established in objective 1. However, this was not the case with SHRI and SHRIS. Specifically, when the performance of the simpler model (SHRI) was compared to that of the more complex one (SHRIS), similar overall performance was observed for both models wherein both models had close mean difference and mean squared difference (see Table 1 SHRI and SHRIS models).

Hence SPRI appeared to have better performance than the other models and increasing the level of complexity by going from SPRI to SPRIS did not improve on the accuracy of the estimates that were generated under SPRI. Hence, these results lead to the conclusion that modeling the slope as a random effect along with the intercept did not improve the performance of the associated models (SHRIS and SPRIS) compared to the simpler models that assume solely random intercept (SHRI and SPRI).

*Objective 4* Our bootstrap pseudo simulation study also aimed at investigating the effect of multivariate modeling versus the univariate implementation of the models that assume that the outcomes are independent. In this regard a comparison was undertaken between the performance of the RI model and that of SHRI and SPRI, and between RIS and that of SHRIS and SPRIS (Tables 1, 3). The performance of SHRI and RI did not significantly differ since their associated mean difference and mean squared differences were close (see Table 1). Hence, this observation indicates that under models with random intercept, the multivariate shared model (SHRI) did not improve the accuracy of the estimates compared to those generated under the univariate random intercept model (RI). However, this conclusion did not hold when we compared SPRI and RI. Specifically, we noticed that 66% of the estimates generated under SPRI had a 42% average reduction in mean difference compared to those estimates generated under RI (see Table 3 column a, and footnote Table 3). For instance, 77 and 73% reductions in mean difference were observed for the slope estimates of the outcomes creatinine and eGFR generated under SPRI compared to those generated under RI. Nonetheless, the mean squared differences did not greatly differ in the estimates of these two models.

When SHRIS and RIS were compared, a minimal reduction (only 4%) was denoted for the mean squared differences in the majority of the estimates (about 80%) that were generated under SHRIS compared to RIS; but the mean differences did not differ between the 2 models. Hence SHRIS and RIS exhibited similar performance. Compared to RIS, about 50% of the estimates generated under SPRIS exhibited an observed reduction in the mean difference by about 41% on average (see Table 3 column b, and footnote Table 3), while the mean squared difference did not greatly differ between the 2 models. These results suggest that multivariate modeling specifically SPRI and SPRIS had an advantage in performance specifically in Mean Difference compared to the univariate models RI and RIS respectively (Table 3). However, this conclusion did not hold for the shared multivariate models SHRI and SHRIS wherein their associated mean difference and mean squared difference did not greatly differ from those under the univariate RI and RIS respectively. Hence only the separate multivariate models (SPRI and SPRIS) exhibited improved performance over their univariate counterpart models RI and RIS. This result also endorses our conclusion in objective 2 which stated that separate random effect models (SPRI and SPRIS) had better performance compared to the shared random effect models (SHRI and SHRIS).

## Motivating example: renal transplant application

The current study was motivated by the cohort of renal transplant patients. The dataset consisted of 110 patients who underwent renal transplantation in the year 2000 and were followed for 3 years (till year 2003) post-transplant until they experience graft rejection or end of study period. After the scheduled termination date of the study in year 2003, patients were no longer followed and assessment of their kidney function was not undertaken afterwards.

In case of graft rejection, patients had to revert back to the dialysis regimen and were not monitored afterwards. About 19% of the patients in this cohort experienced graft rejection. Renal function and progression of renal disease is assessed by 3 correlated markers, BUN, serum Cr, and eGFR. These outcomes follow the lognormal distribution. In this study BUN and Cr had a positive correlation of 0.624 (*P* value <0.0001), while BUN and eGFR had a negative correlation of −0.63 (P value <0.0001) and that of Cr and eGFR was −0.71 (P value <0.0001). Changes in the levels of markers are what usually determine the progression of renal disease. In this regard, decrease in the level of BUN and Cr indicates an improvement in the kidney function over time. However, the opposite is true for eGFR where an increase in the levels of this marker indicates improved kidney function. This explains the positive correlation between BUN and Cr, and the negative ones between these markers and eGFR. The normal values for BUN are between 8 and 25 mg per 100 mL of blood, for Cr the normal range is between 0.7 and 1.3 mg per 100 mL of blood, and for eGFR this range is approximately between 90 and 120 mL/min/1.73 m^2^ for men and women respectively. Measurements on the three markers were taken on every patient for a 3-year period starting with the baseline measure pre-transplant registered as month 0, then at months 1, 3, 6, 12, 24, and 36 post-transplant.

The four models examined in this study were applied on this cohort of patients and the objective was to estimate the slope for every outcome over time along with some associated key covariates that include patient’s age, gender, and race and the vital status (deceased or living) of the respective donor along with some basic demographics such as donor’s gender and race (see Table 4). Applying the mGLMM on this cohort of patients enabled us to assess the effect of these various covariates on all the renal markers (BUN, creatinine and eGFR) collectively through the joint modeling approach. Moreover, the random effects term in the mGLMM accounts for the correlations between the repeated measures for every patient and the different markers respectively, and for the between-patients variation in the length of stay and completeness of measurements of renal outcomes. This variation emerges between patients who may dropout before the intended termination date of the study. In addition, the link functions and the generalized approach available in mGLMM make it feasible to model the renal outcomes measured in this study and which were found to follow the lognormal distribution without necessitating any transformation to achieve normality.

**Table 4:** Application of the four multivariate models on the renal transplant dataset with all covariates

Model	Outcome	Slope African_America					
		Intercept (SE)	Slope time (SE)	Slope male (SE)	n (SE)	Slope age (SE)	Slope donor male (SE)
		P-value	P-value	P-value	P-value	P-value	P-value
SHRI	BUN	3.08890 (0.08067)	−0.01995 (0.00203)	0.17280 (0.04095)	0.08899 (0.04102)	0.004602 (0.00147)	−0.07432 (0.04300)
		<0.0001	<0.0001	<0.0001	0.0301	0.0018	0.0841
	Creatinine	0.85950 (0.11300)	−0.04514 (0.00339)	0.24650 (0.05708)	0.23420 (0.05718)	−0.001980 (0.00205)	−0.15420 (0.05993)
		<0.0001	<0.0001	<0.0001	<0.0001	0.3336	0.0102
	eGFR	3.38800 (0.13700)	0.05333 (0.00431)	0.01762 (0.06910)	−0.05963 (0.06923)	−0.00249 (0.00248)	0.16960 (0.07255)
		<0.0001	<0.0001	0.7988	0.3891	0.3163	0.0195
SHRIS	BUN	3.09780 (0.07982)	−0.01986 (0.00204)	0.16890 (0.04069)	0.07361 (0.04073)	0.00461 (0.00146)	−0.07389 (0.04272)
		<0.0001	<0.0001	<0.0001	0.0709	0.0017	0.0838
	Creatinine	0.86840 (0.11250)	−0.04505 (0.00340)	0.24270 (0.05697)	0.21880 (0.05707)	−0.00198 (0.00204)	−0.15380 (0.05982)
		<0.0001	<0.0001	<0.0001	0.0001	0.3347	0.0102
	eGFR	3.39690 (0.13820)	0.05342 (0.00438)	0.01375 (0.06979)	−0.07501 (0.06992)	−0.00248 (0.00250)	0.17010 (0.07328)
		<0.0001	<0.0001	0.8438	0.2834	0.3226	0.0204
SPRI	BUN	3.08030 (0.10790)	−0.01904 (0.00194)	0.17130 (0.05497)	0.10870 (0.05506)	0.00486 (0.00198)	−0.08662 (0.05774)
		<0.0001	<0.0001	0.0019	0.0486	0.0142	0.1337
	Creatinine	0.85050 (0.12430)	−0.04406 (0.00333)	0.24560 (0.06278)	0.25580 (0.06288)	−0.00172 (0.00226)	−0.16780 (0.06594)
		<0.0001	<0.0001	<0.0001	<0.0001	0.4478	0.011
	eGFR	3.41330 (0.14630)	0.05085 (0.00384)	0.01952 (0.07389)	−0.10570 (0.07401)	−0.00329 (0.00266)	0.20460 (0.07761)
		<0.0001	<0.0001	0.7917	0.1533	0.216	0.0084
SPRIS^a^	BUN	3.38950 (0.03001)	−0.01857 (0.00215)				
		<0.0001	<0.0001				
	Creatinine	0.94220 (0.04326)	−0.04421 (0.00342)				
		<0.0001	<0.0001				
	eGFR	3.36560 (0.04724)	0.05049 (0.00388)				
		<0.0001	<0.0001				

^a^SPRIS converged for time and time squared covariates only.

By applying the four models we were able to identify the one(s) that had the best fit determined by measures such as AIC, BIC and −2 log likelihood (see Table 5), and compare the associated standard errors for each model. In addition we applied the univariate random intercept model (RI) and univariate random intercept and slope (RIS) on every outcome separately (see Table 6) to compare the estimates obtained under the multivariate application versus the univariate ones. The multivariate model SPRIS did not converge when all these covariates were included concurrently but just with the covariates time and time square. The relation between time and the three markers appeared to be curvilinear; hence, time squared was also included in the model to account for this non-linear relationship. The fitting measures when the 4 models were applied on the renal dataset with only time and time squared as covariates are also presented in Table 5. The variance–covariance matrices of the multivariate and univariate models are reported in Appendix 1 and Appendix 2, respectively.

**Table 5:** Measures of fit for the multivariate models applied on renal transplant dataset with (a) all covariates (b) time and time squared

	Model	AIC	BIC	−2logl
(a) All covariates	SHRI	4,000.21	4,010.98	3,992.21
	SHRIS	3,999	4,015.14	3,987
	SPRI	3,358.24	3,382.46	3,340.24
	SPRIS^a^	(–)	(–)	(–)
(b) Time and time squared	SHRI	4,040.59	4,051.39	4,032.59
	SHRIS	4,036.7	4,052.91	4,024.7
	SPRI	3,732	3,756.64	3,714.34
	SPRIS^a^	3,749.12	3,808.53	3,705.12

^a^SPRIS model converged for only Time and Time Squared covariates.

**Table 6:** Application of the two univariate models on the renal transplant dataset with all covariates

Model	Outcome	Intercept (SE)	Slope time (SE)	Slope male (SE)	Slope AA (SE)	Slope age (SE)	Slope donor male (SE)
		P-value	P-value	P-value	P-value	P-value	P-value
RI	BUN	3.08470 (0.10610)	−0.01942 (0.00195)	0.17290 (0.05408)	0.09597 (0.05416)	0.00475 (0.00195)	−0.08116 (0.05679)
		<0.0001	<0.0001	0.0015	0.0768	0.0148	0.1534
	Creatinine	0.85850 (0.11920)	−0.04527 (0.00341)	0.24620 (0.06037)	0.23670 (0.06047)	−0.00199 (0.00217)	−0.15380 (0.06338)
		<0.0001	<0.0001	<0.0001	<0.0001	0.3582	0.0155
	eGFR	3.40210 (0.13990)	0.05221 (0.00394)	0.01729 (0.07089)	−0.08284 (0.07101)	−0.00289 (0.00255)	0.18690 (0.07443)
		<0.0001	<0.0001	0.8074	0.2438	0.2568	0.0123
RIS	BUN	3.09910 (0.10370)	−0.01936 (0.00213)	0.16680 (0.05290)	0.07354 (0.05297)	0.00468 (0.00191)	−0.07877 (0.05556)
		<0.0001	<0.0001	0.0017	0.1655	0.0141	0.1567
	Creatinine	0.83970 (0.11780)	−0.04528 (0.00342)	0.24670 (0.06001)	0.23940 (0.06007)	−0.00163 (0.00216)	−0.15450 (0.06298)
		<0.0001	<0.0001	<0.0001	<0.0001	0.4501	0.0144
	eGFR	3.42390 (0.13840)	0.05222 (0.00395)	0.01660 (0.07050)	−0.08582 (0.07058)	−0.00332 (0.00253)	0.18760 (0.07401)
		<0.0001	<0.0001	0.8139	0.2244	0.1906	0.0115

**Table 7:** Variance–covariance matrix of SHRI and SHRIS when applied on renal transplant dataset with all covariates

Model	Outcome	Variance intercept (SE)	Covariance intercept, slope	Variance slope (SE)	Variance residuals (SE)
SHRI		0.02077 (0.005809)			
	BUN				0.1494 (0.009515)
	Creatinine				0.4197 (0.02320)
	eGFR				0.6791 (0.03934)
SHRIS		0.01562 (0.007302)	0.000242 (0.000277)	0.000016 (0.00002)	
	BUN				0.1431 (0.0114)
	Creatinine				0.4151 (0.02342)
	eGFR				0.6929 (0.04286)

**Table 8:** Variance–covariance matrix of SPRI when applied on renal transplant dataset with all covariates

	Outcome	Variance intercept (SE)	Covariance intercepts BUN, Cr (SE)	Covariance intercepts BUN, eGFR (SE)	Covariance intercepts Cr, eGFR (SE)	Variance Residuals (SE)
SPRI	BUN	0.05856 (0.01151)	0.0666 (0.01145)	−0.07767 (0.01343)	−0.1188 (0.01764)	0.1345 (0.007824)
	Creatinine	0.04257 (0.01542)				0.4102 (0.02357)
	eGFR	0.06204 (0.02132)				0.5464 (0.0314)

**Table 9:** Variance–covariance matrix of SPRIS when applied on renal transplant dataset with covariates time and time squared

SPRIS^a^	Intercept	Intercerpt	Intercept	Slope	Slope	Slope
	BUN (SE)	Cr (SE)	eGFR (SE)	BUN (SE)	Cr (SE)	eGFR (SE)
Intercept	0.2606					
BUN (SE)	(0.02609)					
Intercerpt	0.2932	0.1229				
Cr (SE)	(0.04267)	(0.05179)				
Intercept	−0.3064	−0.1173	<0.000001			
eGFR (SE)	(0.04685)	(0.05906)	(–)			
Slope	−0.00231	0.00442	−0.00964	0.006384		
BUN (SE)	(0.002248)	(0.004197)	(0.1256)	0.1897		
Slope	−0.0047	0.003245	−0.00905	0.005992	<0.000001	
Cr (SE)	(0.002801)	(0.004418)	(0.118)	(0.1781)	(0.002482)	
Slope	0.005316	−0.00378	0.009299	−0.00616	<0.000001	<0.000001
eGFR (SE)	(0.003132)	(0.005003)	(0.1212)	(0.183)	(0.002792)	(–)

^a^SPRIS converged for only time and time squared covariates.

**Table 10:** Variance–covariance matrix of RI and RIS when applied on renal transplant dataset with all covariates

Model	Outcome	Variance intercept (SE)	Covariance intercept, slope (SE)	Variance slope (SE)	Variance residuals (SE)
RI	BUN	0.05553 (0.01111)			0.1352 (0.007904)
	Creatinine	0.03038 (0.01423)			0.4205 (0.02455)
	eGFR	0.04494 (0.01956)			0.5599 (0.03268)
RIS	BUN	0.0543 (0.01257)	−0.00049 (0.000564)	0.00012 (0.000038)	0.1166 (0.007530)
	Creatinine	0.01192 (0.02067)	0.000865 (0.000829)	<0.000001 (–)	0.4211 (0.02463)
	eGFR	0.02113 (0.02826)	0.001116 (0.001122)	<0.000001 (–)	0.5605 (0.03278)

As an overall discussion of the estimates we can deduce that for all multivariate models (see Table 4) patients appear to have improved kidney function over time as evidenced by the negative slopes of BUN and Cr, and positive ones for eGFR. This improvement was demonstrated by the estimated decrease of 1.02 mg per 100 mL of blood in BUN on average across all multivariate models and 1.1 mg per 100 mL of blood for Cr. Along the same line, eGFR levels were estimated to increase by an average across all multivariate models of 1.1 mL/min/1.73 m^2^. This significant improvement in the progression of renal disease over time detected in the multivariate models was also established in univariate models (see Table 6). Gender appeared to be significantly associated with the changes in BUN and Cr but not with eGFR, and males patients were shown to have inferior kidney function compared to females. This conclusion was demonstrated in the multivariate models with the attributed increase in BUN levels for males versus females of 1.2 mg per 100 mL of blood and of 1.3 mg per 100 mL of blood for Cr (see Table 4). A similar conclusion was reached under the univariate models (see Table 6). This detected increase in the levels of these markers in males compared to females led to the conclusion that female patients have better prognosis of kidney disease compared to males that could be attributed to the protective effect of estrogen [23].

Race was found to be associated with BUN and Cr in the SHRI and SPRI models and only Cr in SHRIS. In the univariate models (see Table 6), race was found to be associated only with Cr and, unlike the multivariate models, no association was detected with BUN. Specifically, non-African American patients appeared to be at a disadvantage compared to other races due to their 1.1 mg per 100 mL of blood increase in BUN levels on average and 1.3 mg per 100 mL of blood increase on average in Cr (see Table 4). This conclusion was also valid under the univariate models; nevertheless, it was just demonstrated with Cr.

Age appeared to be positively correlated with BUN in all models and with every 1 year increase in age of transplantation corresponds to an increase in BUN levels of approximately 1.6 mg per 100 mL of blood on average. Hence those patients who had their renal transplantation at older ages were at a disadvantage in terms of renal function and progression of kidney disease compared to those patients who had the transplant at younger ages. This conclusion holds for all models had it be multivariate or univariate.

Males were better donors than females since patients who received their kidneys from male donors exhibited a decrease of approximately 1.2 mg per 100 mL of blood in their Cr levels, and an increase of approximately 1.2 mL/min/1.73 m^2^ in eGFR levels compared to patients who had female donors (Tables 4, 6). This result is in line with other studies that showed females to be poorer donors than males, an observation attributed to nephron underdosing and hence nephron overload in females [23–25]. No association was detected between the donor’s race and BUN in all models. The other covariates such as donor’s gender and vital status were found to be not associated with any of the outcomes and as a result were not included in any of the models.

## Comparison of models fit

SPRI exhibited the best fit as demonstrated by its lowest associated pseudo AIC, BIC and −2log l (see Table 5) wherein average decreases of 641, 631 and 650 in these measures were denoted between SPRI compared to the other models SHRI and SHRIS. Compared to the univariate model RI, the standard errors generated under SPRI were mostly slightly larger than the ones generated under RI. However, this was not the case when we compared the standard errors under SHRI and those under the univariate model RI wherein all the estimates under SHRI had smaller SE than those under RI. The same conclusion was observed when we compared SHRIS to RIS and we denoted that all estimates under SHRIS had smaller standard errors than those with RIS. This conclusion was anticipated since only one additional parameter (variance of the intercept) needed to be estimated under SHRI as indicated in the variance–covariance matrix (see Appendix 1). The same case was denoted for RI where also one additional parameter (variance of the intercept) needed to be estimated for every outcome (see Appendix 2) leading to three additional parameters that pertain to the three outcomes. The same can be said for SHRIS and RIS models. However SPRI had additional six parameters in the variance–covariance matrix (see Appendix 1). A comparison of the four multivariate models based on the application to the renal data with time and time squared as covariates (see Table 5), SPRI still exhibited the best fit and its associated AIC, BIC and −2log l were the smallest compared to SHRI, SHRIS and SPRIS.

## Discussion

In this study we demonstrate a novel application of the different mGLMM approaches to a renal transplant dataset characterized by its small sample size of 110 patients and correlated multiple outcomes with lognormal distribution. While mGLMM is based on multivariate modeling of the correlated outcomes via joint modeling of latent variables, other approaches have also been used. For example, earlier analysis of the same renal transplant data [26] was based on a Two-stage likelihood-based approach wherein the individual observations for every patient are reduced into slopes computed in stage 1. A jointly modeling of individual slopes and their correlations along with the number of visits for every patient is conducted in stage 2. Maximization of the likelihood function that is integrated over the random effects results in maximum likelihood estimates of the population slopes and empirical Bayes estimates for the individual slopes. Since individual observations for every patient are reduced to the individual slopes then this required imposing an assumption that only patients with two visits or more can be included in the study. Moreover, it was assumed that all sufficient information is contained in the slopes and that the intercept could be discarded and excluded from the likelihood function. These two assumptions are no longer required in the mGLMM approaches presented in this study since intercepts were incorporated as a shared random effect or separate ones and individual observations are modeled rather than their corresponding slopes, hence the restriction of eligibility for those with two observations and more was relaxed here. In this study we gave additional insight into the assessment of shared versus separate models in clinical settings of small sample size. Our current assessment also includes the level of computational complexity and accuracy of the different models. As expected, there has been some discrepancy in the computational burden to fit each of the models. For example, the SPRIS model did not converge when multiple covariates were included along with time and time squared. A natural explanation for this phenomenon is having a large number of 21 parameters in the variance–covariance matrix of SPRIS that needed estimation (Appendix 1) and a small sample size of 110 did not help achieve convergence. This observation highlights a drawback with SPRIS and suggests that SPRIS should be used with caution in settings of small sample size. SPRI had the best fit when applied to the renal dataset and generated the most accurate estimates with minimum mean difference and mean squared differences. This advantage could be due to the fact that the number of parameters in the variance–covariance matrix for SPRI (6 parameters) is midway between the SHRI’s (1 parameter) and SHRIS’ (3 parameters) and that for SPRIS (21 parameters). This result is in agreement with what has been shown in studies with large sample size [22] where SPRI also demonstrated the best fit in terms of smallest AIC, BIC and −2log l. To validate these models using another independent dataset, the mGLMM approach was applied on a longitudinal study of type 1 diabetic patients with large sample size and multiple cardiovascular disease outcomes that have mixed non-normal distributions [27, 28]. Our results showed that SPRI had the best fit in terms of smallest AIC, BIC, and -2log l compared to the other models and had the minimum mean difference and mean difference squared in the majority of the estimates. This confirms again that SPRI had the best performance and best fit in the context of large datasets as was the case with the small dataset.

Models with separate random effects exhibited better performance in terms of mean difference and mean squared differences compared to those with shared ones and also demonstrated superior performance than the univariate RI and RIS models. Nonetheless, increasing the level of complexity from SPRI to SPRIS by adding the slope as a random effect along with the intercept did not increase the level of accuracy; on the contrary convergence problems were encountered. Hence it appears that the SPRI model that is midway in complexity was favored by the renal dataset and the bootstrap study.

## Conclusion

In the context of a relatively small sample size study, we were able to demonstrate that the mGLLMs with SPRIs and separate random intercepts and slopes (SPRIS) generated more accurate estimates compared to the SHRI and SHRIS models. Similar conclusion was reached when we compared SPRI and SPRIS to their univariate alternatives RI and RIS. Convergence issues were encountered with SPRIS when the number of covariates increased, however, SPRI had the best fit and most accurate performance compared to the other models and its successful convergence was consistently achieved.

## Appendix 1

See Tables 7, 8, 9.

## Appendix 2

See Table 10.

## Abbreviations

BUN: blood urea nitrogen
Cr: serum creatinine
eGFR: estimated glomerular filtration rate
mGLMM: multivariate generalized linear mixed model
MLIRT: multilevel item response theory
MLMM: multivariate linear mixed model
SHRI: shared random intercept
SHRIS: shared random intercept and slope
SPRI: Separate random intercept
SPRIS: separate random intercept and slope

## References

[1] James GD, Seally JE, Alderman M, Ljungman S, Mueller FB, Pecker MS (1988). A longitudinal study of urinary creatinine and creatinine clearance in normal subjects. Race, sex and age differences. Am J Hypertens.

[2] Schrier RW (2008). Blood urea nitrogen and serum creatinine not married in heart failure. Circ Heart Fail.

[3] Froissart M, Rossert J, Jacquot C, Paillard M, Houillier P (2005). Predictive performance of the modification of diet in renal disease and Cockcroft-Gault equations for estimating renal function. J Am Soc Nephrol.

[4] Verhave JC, Fesler P, Ribstein J, du Cailar G, Mimran A (2005). Estimation of renal function in subjects with normal serum creatinine levels: influence of age and body mass index. Am J Kidney Dis.

[5] Cirillo M, Anastasio P, De Santo NG (2005). Relationship of gender, age, and body mass index to errors in predicted kidney function. Nephrol Dial Transplant.

[6] Sammel M, Lin X, Ryan L (1999). Multivariate linear mixed models for multiple outcomes. Stat Med.

[7] Xu J, Zeger SL (2001). The evaluation of multiple surrogate endpoints. Biometrics.

[8] He B, Luo S (2013) Joint modeling of multivariate longitudinal measurements and survival data with applications to Parkinson’s disease. Stat Methods Med Res (In Press Published on line first on April 16, 2013) 10.1177/0962280213480877 PMC3883896 23592717

[9] Adams R, Wilson M, Wu M (1997). Multilevel item response models: an approach to errors in variables regression. J Educ Behav Stat.

[10] Andersen E (2004). Latent regression analysis based on the rating scale model. Psychol Sci.

[11] Christensen K, Bjorner J, Kreiner S, Petersen J (2004). Latent regression in loglinear Rasch models. Commun Stat Theory Methods.

[12] Mislevy R (1985). Estimation of latent group effects. J Am Stat Assoc.

[13] Zwinderman A (1991). A generalized Rasch model for manifest predictors. Psychometrika.

[14] Maier K (2001). A Rasch hierarchical measurement model. J Educ Behav Stat.

[15] Kamata A (2001). Item analysis by the hierarchical generalized linear model. J Educ Meas.

[16] Fox J (2005). Multilevel IRT using dichotomous and polytomous response data. Br J Math Stat Psychol.

[17] Pinheiro J, Liu C, Wu Y (2001). Efficient algorithms for robust estimation in linear mixed-effects models using the multivariate t distribution. J Comput Graph Stat.

[18] Fieuws S, Verbeke G (2004). Joint modeling of multivariate longitudinal profiles: pitfalls of the random effect approach. Stat Med.

[19] Fieuws S, Verbeke G, Molenberghs G (2007). Random-effects models for multivariate repeated measures. Stat Methods Med Res.

[20] Bandyopadhyay S, Ganguli B, Chatterjee A (2011). A review of multivariate longitudinal data analysis. Stat Methods Med Res.

[21] Lachos V, Bandyopadhyay D, Dey D (2011). Linear and nonlinear mixed-effects models for censored HIV viral loads using normal/independent distributions. Biometrics.

[22] Gebregziabher M, Zhao Y, Dismuke CE, Axon N, Hunt KJ, Egede LE (2013). Joint modeling of multiple longitudinal cost outcomes using multivariate generalized linear mixed models. Health Serv Outcomes Res Method.

[23] Zeier M, Dohler B, Oplez G, Ritz E (2002). The effect of donor gender on graft failure. J Am Soc Nephrol.

[24] Kasiske BL, Umen JA (1986). The influence of age, sex, race and body habitus on kidney weight in humans. Arch Pathol Lab Med.

[25] Brenner BM, Cohen RA, Milford EL (1992). In renal transplantation, one size may not fit all. J Am Soc Nephrol.

[26] Jaffa MA, Woolson RF, Lipsitz SR (2011). Slope estimation for bivariate longitudinal outcomes adjusting for informative right censoring by using a discrete survival model: application to the renal transplant cohort. J Royal Stat Soc A.

[27] EDIC Research Group (1999). Epidemiology of diabetes interventions and complications (EDIC): design, implementation and preliminary results of long-term follow up of diabetes control and complications trial cohort. Diabetes Care.

[28] The DCCT Research Group (1986). The diabetes control and complications trial (DCCT): design and methodologic considerations for the feasibility phase. Diabetes.

